# Association between cardiometabolic index and bone mineral density among adolescents in the United States

**DOI:** 10.3389/fendo.2025.1535509

**Published:** 2025-05-08

**Authors:** Haobiao Liu, Rongqi Xiang, Chenyue Liu, Zhuohang Chen, Yuhang Shi, Yiting Liu, Yan Liu

**Affiliations:** ^1^ Department of Dermatology, The Second Affiliated Hospital of Xi’an Jiaotong University, Xi’an, China; ^2^ Department of Occupational and Environmental Health, School of Public Health, Health Science Center, Xi’an Jiaotong University, Xi’an, China; ^3^ Department of Radiology, The First Affiliated Hospital of Xi’an Jiaotong University, Xi’an, China; ^4^ Department of Epidemiology, School of Public Health, Shanghai Institute of Infectious Disease and Biosecurity, Fudan University, Shanghai, China; ^5^ School of Basic Medical Sciences, Shanghai Jiaotong University, Shanghai, China; ^6^ Faculty of Medicine, Dentistry and Health Sciences, Melbourne School of Population and Global Health, The University of Melbourne, Melbourne, VIC, Australia

**Keywords:** cardiometabolic index, bone mineral density, adolescents, population-based study, NHANES

## Abstract

**Objectives:**

The cardiometabolic index (CMI) serves as a comprehensive metric for evaluating cardiometabolic health, and is correlated with several health outcomes. However, research examining the relationship between CMI and bone mineral density (BMD), particularly in adolescent populations, remains limited and warrants further investigation.

**Methods:**

The weighted multiple linear regression analysis was conducted to elucidate the association between CMI and BMD.

**Results:**

Our study ultimately included 1,514 participants. After adjusting for pertinent covariates, we observed that per-unit increases in the CMI corresponded with reductions in BMD by 0.052 g/cm^2^ for femoral neck (*β*=-0.052, 95% CI: -0.087 to -0.018) and 0.048 g/cm^2^ for lumbar spine (L1-L4) (*β*=-0.048, 95% CI: -0.085 to -0.011). In quartile analyses, individuals in the highest quartile displayed significantly reduced BMD at the femoral neck (*β*=-0.036, 95% CI: -0.064 to -0.007) and lumbar spine (L1-L4) (*β*=-0.041, 95% CI: -0.070 to -0.011) compared to those in the lowest quartile (*P*<0.05). No statistical significance was detected between CMI and BMD at the total femur, trochanter, and intertrochanter sites. Furthermore, stratified analyses indicated no significant interactions involving age, sex, or race in relation to CMI and BMD.

**Conclusions:**

In the adolescent population, CMI is inversely related to BMD. These findings highlight a potential link between cardiometabolic health and bone health. Future longitudinal investigations are warranted to determine causal relationships and underlying mechanisms.

## 1 Introduction

Skeletal health during adolescence plays a crucial role in lifelong well-being. This period marks a critical window for the rapid accrual of bone mineral density (BMD), which largely determines the peak BMD achieved in adulthood and its maintenance over time. Peak BMD has a significant correlation with the risk of developing osteoporosis and experiencing fractures in later life, as fluctuations in BMD can increase the susceptibility to fragility fractures and other bone-related diseases (1). Therefore, understanding the factors influencing BMD during adolescence is of paramount clinical importance for preventing osteoporosis and promoting optimal skeletal health.

In recent years, the potential relationship between cardiometabolic health indicators and bone health has gained significant attention, particularly in pediatric and adolescent populations. The cardiometabolic index (CMI) is a composite marker that integrates both lipid profiles and central obesity to assess cardiometabolic risk. CMI is calculated as triglyceride (TG) to high-density lipoprotein cholesterol (HDL-C) ratio multiplied by waist-to-height ratio (WHtR). This index has been widely used to evaluate metabolic dysfunction and cardiovascular disease risk. However, its relationship with bone health remains underexplored. Given that dysregulated lipid metabolism and obesity-related inflammation are known to influence bone metabolism, CMI may serve as a valuable indicator for assessing the relationship between cardiometabolic health and BMD.

Lipid metabolism plays a key role in skeletal health. For instance, elevated TG levels have been linked to lower BMD in older adults, revealing an inverse association between lipid profiles and BMD (2). Lipidomics profiling also indicates significant alterations in lipid composition among individuals with low BMD compared to those with normal BMD (3). Additionally, a Mendelian randomization study supports the causal relationship between lipid metabolites and bone health (4). Furthermore, obesity, a known risk factor for cardiovascular diseases, is closely associated with changes in bone quality. Studies have indicated that excessive adipose tissue may secrete adipokines such as leptin and adiponectin, which can influence bone metabolism and remodeling (5). The accumulation of visceral fat during early life negatively impacts bone health, consequently elevating the risk of osteopenia and osteoporosis in adults and the elderly (6).

Although the relationship between cardiometabolic health and conditions such as depression (7), non-alcoholic fatty liver disease (8), and chronic obstructive pulmonary disease (9) has been explored, there is a scarcity of research focusing on its association with BMD, particularly in adolescent populations. Adolescence is not only a pivotal period for BMD accrual but also a time of significant physiological changes in the cardiometabolic system, necessitating further investigation in this unique cohort. During this developmental stage, bone metabolism is particularly sensitive to nutritional, hormonal, and cardiometabolic factors. Understanding the influence of cardiometabolic health on BMD during adolescence will provide valuable insights into the early prevention of chronic conditions such as osteoporosis, cardiovascular diseases, and metabolic syndrome. Moreover, elucidating the link between CMI and BMD may help determine whether CMI can serve as a potential indicator for skeletal health assessment in adolescents.

Against this backdrop, the present study aims to evaluate the association between CMI and BMD in adolescents, addressing an existing gap in the literature and providing new insights into the potential role of CMI as an alternative indicator for skeletal health.

## 2 Materials and methods

### 2.1 Study design and population

Information was extracted from the NHANES spanning from 2005 to 2010, which involved 4,865 participants within the age range of 12 to 19. Following this, individuals without BMD information (*n*=711), those lacking complete CMI data or presenting abnormal values (*n*=36), and participants with missing covariate information (*n*=158) were excluded from the analysis. Ultimately, this resulted in the inclusion of 1,514 participants in the study (**Supplementary Figure S1**).

### 2.2 Evaluation of CMI

CMI was derived from anthropometric and biochemical measurements, specifically height, waist circumference (WC), TG, and HDL-C. TG and HDL-C were measured using an enzymatic method on a Roche Modular P Chemistry Analyzer (Roche Diagnostics, Indianapolis, IN, USA). Quality control procedures followed the guidelines of the National Center for Health Statistics. Initially, the WHtR was computed as the ratio of WC to height. Subsequently, CMI was calculated by multiplying the WHtR by the ratio of TG to HDL-C, as shown in the formula below.


CMI=(TG/HDL-C)×WHtR

### 2.3 Inspection of BMD

In this study, the primary outcome measures were total femur BMD, femoral neck BMD, trochanter BMD, intertrochanter BMD, and lumbar spine (L1-L4) BMD. The femur and spine scans were acquired with a Hologic QDR-4500A dual-energy X-ray absorptiometry (DXA) scanner (Hologic, Inc., Bedford, MA, USA), using software version Discovery v12.4. Typically, lumbar spine BMD was derived from a dedicated lumbar spine scan (vertebrae L1-L4) using DXA. The quality control and calibration procedures were performed using a standardized phantom provided by the manufacturer. For a comprehensive description of the procedures utilized, please consult the Body Composition Procedures Manual (10).

### 2.4 Covariate assessment

All factors considered and adjusted for at present research were gathered in accordance with existing literature (11, 12). These factors encompassed age, sex, race, poverty income ratio (PIR), body mass index (BMI), levels of physical activity, serum biochemistry indicators (total calcium, phosphorus, and total protein), as well as systolic blood pressure, diastolic blood pressure, and glycohemoglobin. See **Supplementary Table S1** for a complete definition and categorization of covariates. Additional information regarding covariates is available on the website (13).

### 2.5 Statistical analysis

Continuous variables were reported as weighted mean values along with their standard errors, whereas categorical variables were presented as unweighted counts along with corresponding weighted percentages. All statistical analyses conducted in this research utilized R software (version 4.2.2), ensuring adherence to NHANES sampling protocols as outlined in the analysis guidelines (13). To compare baseline characteristics across CMI quartiles, we used weighted ANOVA for continuous variables and weighted Rao-Scott chi-square tests for categorical variables. When significant overall differences were detected, we conducted *post-hoc* pairwise comparisons using Bonferroni-corrected methods. To investigate the relationship between CMI and BMD, we developed three distinct linear regression models based on specific criteria for variable selection. Model 1 was adjusted solely for BMI, as it is closely related to weight and health status and has been shown to significantly influence BMD. Model 2 further accounted for age, sex, and race, which are known to have significant associations with BMD and can impact CMI expression across diverse populations. Finally, Model 3 included additional adjustments for PIR, physical activity, serum biochemical indicators, systolic blood pressure, diastolic blood pressure, and glycohemoglobin. These variables were selected based on biological plausibility and previous research findings. We reported the coefficient of determination (R^2^) for the weighted regression model to assess the proportion of variance in BMD explained by the independent variables. To compare CMI with traditional lipid indicators, including HDL-C, low-density lipoprotein cholesterol (LDL-C), LDL-C and HDL-C ratio, and total cholesterol (TC), we conducted additional multiple linear regression analyses, where each indicator was separately included in the model with the same set of covariates. The absolute values of standardized *β* coefficients were compared across models to evaluate their relative associations with BMD. Subgroup and interaction analyses were performed according to age, sex, and race. Furthermore, sensitivity analysis was further performed to test the robustness of this association. We additionally adjusted other covariates such as alanine aminotransferase, aspartate aminotransferase, alkaline phosphatase, blood urea nitrogen, and uric acid, and re-analysis the results using unweighted data. Statistical significance was set as *P*<0.05.

## 3 Result

### 3.1 Basic characteristics of the study population

The present study included a total of 1,514 adolescents aged 12 to 19 years. The weighted characteristics of participants were compared at different CMI levels, and the results are detailed in **Table 1**.

**Table 1:** Weighted characteristics of participants based on cardiometabolic index quartiles.

Characteristic	Cardiometabolic index					*P* value
	Total	Q1	Q2	Q3	Q4	
Age, years	15.49 (2.21)	14.94 (2.06)^b,c^	15.36 (2.28)^d^	15.96 (2.08)^b,d^	15.72 (2.30)^c^	<0.001
Sex						0.706
Male	841 (53.35)	231 (52.04)	206 (51.28)	180 (52.90)	224 (57.21)	
Female	673 (46.65)	179 (47.96)	165 (48.72)	164 (47.10)	165 (42.79)	
Race						<0.001
Non-Hispanic White	463 (63.02)	91 (51.58)^a,b,c^	112 (61.53)^a,d,e^	126 (71.33)^b,d,f^	134 (67.67)^c,e,f^	
Non-Hispanic Black	400 (13.83)	171 (24.11)^a,b,c^	98 (13.97)^a,d,e^	81 (10.55)^b,d,f^	50 (6.60)^c,e,f^	
Hispanic	571 (16.56)	122 (14.11)^a,c^	133 (15.67)^a,d,e^	122 (13.84)^d,f^	194 (22.69)^c,e,f^	
Other	80 (6.60)	26 (10.21)^a,b,c^	28 (8.83)^a,d,e^	15 (4.28)^b,d,f^	11 (3.04)^c,e,f^	
Poverty income ratio						0.010
<1.3	614 (27.11)	162 (25.33)^b,c^	139 (24.47)^e^	131 (22.90)^b,f^	182 (35.81)^c,e,f^	
1.3-3.5	548 (35.07)	140 (33.28)^a,b,c^	143 (35.32)^a^	123 (35.34)^b^	142 (36.36)^c^	
>3.5	352 (37.82)	108 (41.39)^c^	89 (40.21)^e^	90 (41.75)^f^	65 (27.82)^c,e,f^	
Physical activity						0.458
Low	466 (28.24)	112 (25.37)	107 (26.48)	111 (26.87)	136 (34.30)	
Medium	204 (12.89)	58 (14.24)	46 (10.76)	56 (14.83)	44 (11.71)	
High	844 (58.87)	240 (60.39)	218 (62.77)	177 (58.30)	209 (53.99)	
Body mass index, Kg/m^2^	23.12 (5.25)	20.34 (2.83)^a,b,c^	22.09 (4.53)^a,d,e^	23.44 (4.52)^b,d,f^	26.64 (6.35)^c,e,f^	<0.001
Total calcium, mg/dL	9.66 (0.30)	9.66 (0.29)	9.65 (0.29)	9.64 (0.30)	9.68 (0.32)	0.409
Phosphorus, mg/dL	4.38 (0.67)	4.43 (0.61)^b^	4.48 (0.63)	4.23 (0.70)^b^	4.39 (0.72)	0.001
Total protein, g/dL	7.19 (0.41)	7.21 (0.41)	7.19 (0.41)	7.13 (0.43)	7.22 (0.39)	0.238
Systolic blood pressure, mmHg	109.23 (9.92)	108.35 (9.93)^c^	107.39 (9.74)^d,e^	109.73 (9.12)^d^	111.47 (10.41)^c,e^	<0.001
Diastolic blood pressure, mmHg	59.96 (10.35)	59.89 (9.70)	59.51 (10.45)	59.59 (10.74)	60.86 (10.47)	0.425
Glycohemoglobin, %	5.18 (0.51)	5.20 (0.58)	5.15 (0.29)	5.16 (0.36)	5.20 (0.70)	0.618
Total femur BMD, g/cm^2^	0.983 (0.161)	0.958 (0.155)^c^	0.967 (0.167)	0.999 (0.146)	1.010 (0.168)^c^	0.003
Femoral neck BMD, g/cm^2^	0.906 (0.151)	0.878 (0.146)^b,c^	0.898 (0.160)	0.920 (0.136)^b^	0.928 (0.155)^c^	0.002
Trochanter BMD, g/cm^2^	0.772 (0.139)	0.755 (0.135)^b,c^	0.761 (0.143)	0.786 (0.130)^b^	0.787 (0.147)^c^	0.010
Intertrochanter BMD, g/cm^2^	1.126 (0.186)	1.096 (0.180)^b,c^	1.105 (0.193)	1.145 (0.170)^b^	1.159 (0.193)^c^	0.002
Lumbar spine (L1-L4) BMD, g/cm^2^	0.945 (0.159)	0.928 (0.154)^b,c^	0.929 (0.169)	0.960 (0.147)^b^	0.963 (0.161)^c^	0.024
Triglyceride, mmol/L	0.912 (0.448)	0.514 (0.129)^a,b,c^	0.718 (0.148)^a,d,e^	0.963 (0.206)^b,d,f^	1.460 (0.474)^c,e,f^	<0.001
HDL-C, mmol/L	1.396 (0.311)	1.661 (0.311)^a,b,c^	1.463 (0.246)^a,d,e^	1.318 (0.221)^b,d,f^	1.140 (0.191)^c,e,f^	<0.001
Waist-to-height ratio	0.482 (0.077)	0.439 (0.040)^a,b,c^	0.467 (0.063)^a,d,e^	0.484 (0.064)^b,d,f^	0.539 (0.092)^c,e,f^	<0.001
Cardiometabolic index	0.351 (0.246)	0.137 (0.032)^a,b,c^	0.227 (0.026)^a,d,e^	0.350 (0.044)^b,d,f^	0.694 (0.248)^c,e,f^	<0.001

The numbers of participants in each category are unweighted observed frequencies, while means, standard errors, and percentages are population-weighted. The *post-hoc* pairwise comparisons between quartiles with statistically significant differences are indicated using the following markers: ^a^for the comparison between Q1 and Q2, ^b^for the comparison between Q1 and Q3, ^c^for the comparison between Q1 and Q4, ^d^for the comparison between Q2 and Q3, ^e^for the comparison between Q2 and Q4, ^f^for the comparison between Q3 and Q4. BMD, bone mineral density; HDL-C, high-density lipoprotein cholesterol.

The mean age of all individuals was 15.49 (± 2.21) years, with 53.35% being males and 63.02% identified as non-Hispanic White. Notable disparities in age, race, PIR, BMI, phosphorus, systolic blood pressure, total femur BMD, femoral neck BMD, trochanter BMD, intertrochanter BMD, and lumbar spine (L1-L4) BMD were observed among the different CMI groups (*P*<0.05). The higher group had both an older mean age and a greater proportion of non-Hispanic White individuals in contrast to the lower group. Furthermore, the Q4 group had elevated BMI and systolic blood pressure values, as well as higher BMD levels for the total femur, femoral neck, trochanter, intertrochanter, and lumbar spine (L1-L4) when compared to the Q1 group, whereas the Q1 group exhibited higher PIR and phosphorus levels (*P*<0.05). *Post-hoc* analyses revealed that significant differences in BMD were primarily found between Q1 and Q4 across all sites, and between Q1 and Q3 in the other four sites, except for the total femur.

### 3.2 Association between CMI and BMD among adolescents

Weighted linear regression models were adopted to elucidate the correlation between CMI and BMD among adolescents. In Model 1, adjustment of BMI, we found that each unit increase in CMI was significantly associated with decreased BMD at all measured sites (*P*<0.05). Model 2, which included additional adjustments for age, sex, and race, reaffirmed the existence of a statistically significant correlation (*P*<0.05). When we extended our analysis in Model 3 to include adjustments for PIR, physical activity, total calcium, phosphorus, total protein, systolic blood pressure, diastolic blood pressure, and glycohemoglobin, we observed that per-unit increases in the CMI corresponded with reductions in BMD by 0.052 g/cm^2^ for femoral neck (*β*=-0.052, 95% CI: -0.087 to -0.018) and 0.048 g/cm^2^ for lumbar spine (L1-L4) (*β*=-0.048, 95% CI: -0.085 to -0.011). The weighted regression models explained 39.87% (R^2^ = 0.3987) of the variance in femoral neck BMD and 51.42% (R^2^ = 0.5142) in lumbar spine (L1-L4) BMD. For further details, please see **Table 2**.

**Table 2:** Association between cardiometabolic index and bone mineral density.

Variable	Model 1 *β* (95% CI)	Model 2 *β* (95% CI)	Model 3 *β* (95% CI)
Total femur BMD			
Continuous variable	-0.061 (-0.104, -0.019)**	-0.054 (-0.094, -0.015)**	-0.035 (-0.076, 0.005)
Categorical variable			
Q1	Reference	Reference	Reference
Q2	-0.018 (-0.047, 0.011)	-0.018 (-0.045, 0.009)	-0.012 (-0.038, 0.013)
Q3	-0.007 (-0.033, 0.019)	-0.018 (-0.043, 0.007)	-0.016 (-0.041, 0.009)
Q4	-0.044 (-0.077, -0.010)*	-0.039 (-0.071, -0.008)*	-0.027 (-0.060, 0.006)
Femoral neck BMD			
Continuous variable	-0.072 (-0.107, -0.037)***	-0.066 (-0.099, -0.034)***	-0.052 (-0.087, -0.018)**
Categorical variable			
Q1	Reference	Reference	Reference
Q2	-0.008 (-0.036, 0.020)	-0.008 (-0.035, 0.019)	-0.003 (-0.028, 0.022)
Q3	-0.008 (-0.033, 0.016)	-0.016 (-0.040, 0.008)	-0.015 (-0.041, 0.011)
Q4	-0.049 (-0.077, -0.020)**	-0.045 (-0.071, -0.019)**	-0.036 (-0.064, -0.007)*
Trochanter BMD			
Continuous variable	-0.055 (-0.092, -0.017)**	-0.053 (-0.089, -0.016)**	-0.033 (-0.070, 0.004)
Categorical variable			
Q1	Reference	Reference	Reference
Q2	-0.013 (-0.041, 0.015)	-0.013 (-0.039, 0.014)	-0.008 (-0.033, 0.016)
Q3	-0.003 (-0.024, 0.018)	-0.011 (-0.031, 0.010)	-0.010 (-0.030, 0.011)
Q4	-0.036 (-0.066, -0.007)*	-0.035 (-0.062, -0.008)*	-0.023 (-0.051, 0.005)
Intertrochanter BMD			
Continuous variable	-0.065 (-0.115, -0.015)*	-0.053 (-0.099, -0.008)*	-0.031 (-0.078, 0.015)
Categorical variable			
Q1	Reference	Reference	Reference
Q2	-0.022 (-0.056, 0.011)	-0.022 (-0.053, 0.008)	-0.015 (-0.044, 0.013)
Q3	-0.008 (-0.039, 0.024)	-0.021 (-0.051, 0.008)	-0.018 (-0.047, 0.011)
Q4	-0.048 (-0.087, -0.008)*	-0.040 (-0.078, -0.003)*	-0.025 (-0.064, 0.014)
Lumbar spine (L1-L4) BMD			
Continuous variable	-0.083 (-0.118, -0.047)***	-0.058 (-0.091, -0.026)***	-0.048 (-0.085, -0.011)*
Categorical variable			
Q1	Reference	Reference	Reference
Q2	-0.028 (-0.057, 0.001)	-0.028 (-0.053, -0.003)*	-0.018 (-0.043, 0.006)
Q3	-0.018 (-0.040, 0.005)	-0.032 (-0.050, -0.014)**	-0.028 (-0.048, -0.008)**
Q4	-0.066 (-0.096, -0.036)***	-0.052 (-0.075, -0.028)***	-0.041 (-0.070, -0.011)**

Model 1, adjusted for BMI; Model 2, adjusted for BMI, age, sex, and race; Model 3, adjusted for BMI, age, sex, race, poverty income ratio, physical activity, total calcium, phosphorus, total protein, systolic blood pressure, diastolic blood pressure, and glycohemoglobin. Results in bold indicate statistical significance. BMI, body mass index; CI, confidence interval; BMD, bone mineral density. Cardiometabolic index quartiles, Q1,<0.184; Q2, 0.184-0.280; Q3, 0.281-0.430; Q4, >0.430. * *P*<0.05; ** *P*<0.01; *** *P*<0.001.

When converting the CMI into a categorical variable, a negative correlation with BMD was also observed (**Table 2**). When the analysis was restricted to the adjustment for BMI only (Model 1), a notable association was observed between the Q4 group and lower BMD at all sites in comparison with the Q1 group. Upon further adjustment for age, sex, and race (Model 2), these associations remained statistically significant, while also demonstrating that the Q2 and Q3 groups exhibited significant associations with lower lumbar spine (L1-L4) BMD. Further adjustments in Model 3 revealed a reduction of lower levels of BMD at all sites, but only a significant correlation of the CMI with femoral neck BMD (Q4, *β*=-0.036, 95% CI: -0.064 to -0.007) and lumbar spine (L1-L4) BMD (Q3, *β*=-0.028, 95% CI: -0.048 to -0.008, and Q4, *β*=-0.041, 95% CI: -0.070 to -0.011). Notably, the relationship with lumbar spine (L1-L4) BMD intensified as CMI levels increased, highlighting a potential gradient effect of the CMI on BMD.

### 3.3 Comparison of CMI with traditional lipid indicators

Compared with traditional lipid indicators (HDL-C, LDL-C, LDL-C/HDL-C, and TC), CMI demonstrated a stronger negative association with femoral neck and lumbar spine (L1-L4) BMD, as indicated by larger absolute *β* values (**Supplementary Table S2**). In total femur and trochanter BMD, CMI had the largest absolute *β* value among all lipid indicators, although it did not reach statistical significance, whereas some traditional lipid variables (LDL-C, LDL-C/HDL-C, and TC) were statistically significant. For intertrochanter BMD, CMI had the second-largest effect size but was not statistically significant, while certain traditional indicators (HDL-C, LDL-C, and TC) were significant. These results suggest that CMI may capture metabolic variations relevant to bone health, potentially more comprehensively than individual lipid indicators in specific skeletal sites.

### 3.4 Subgroup and sensitivity analysis

The stratified analysis results were displayed in **Table 3**, indicating that no significant interactions involving age, sex, or race in relation to CMI and BMD.

**Table 3:** Stratified analyses of the association between cardiometabolic index and bone mineral density.

Characteristic	Total femur BMD		Femoral neck BMD		Trochanter BMD		Intertrochanter BMD		Lumbar spine (L1-L4) BMD	
	*β* (95% CI)	*P*_interaction_	*β* (95% CI)	*P*_interaction_	*β* (95% CI)	*P*_interaction_	*β* (95% CI)	*P*_interaction_	*β* (95% CI)	*P*_interaction_
**Age**		0.935		0.896		0.888		0.989		0.505
12–15 years	-0.029 (-0.076, 0.017)		-0.045 (-0.084, -0.006)		-0.030 (-0.073, 0.013)		-0.018 (-0.074, 0.039)		-0.066 (-0.121, -0.010)	
16–19 years	-0.031 (-0.084, 0.022)		-0.055 (-0.101, -0.009)		-0.028 (-0.081, 0.025)		-0.029 (-0.093, 0.035)		-0.017 (-0.072, 0.038)	
**Sex**		0.710		0.837		0.930		0.487		0.914
Male	-0.061 (-0.110, -0.011)		-0.080 (-0.126, -0.035)		-0.061 (-0.106, -0.015)		-0.056 (-0.113, 0.002)		-0.062 (-0.102, -0.022)	
Female	-0.032 (-0.098, 0.035)		-0.038 (-0.090, 0.014)		-0.023 (-0.085, 0.039)		-0.034 (-0.112, 0.044)		-0.048 (-0.112, 0.015)	
**Race**		0.372		0.509		0.424		0.365		0.182
Non-Hispanic White	-0.023 (-0.075, 0.029)		-0.047 (-0.092, -0.003)		-0.027 (-0.074, 0.020)		-0.008 (-0.071, 0.054)		-0.033 (-0.080, 0.015)	
Non-Hispanic Black	-0.061 (-0.173, 0.050)		-0.072 (-0.156, 0.011)		-0.051 (-0.153, 0.050)		-0.068 (-0.192, 0.056)		-0.086 (-0.194, 0.021)	
Hispanic	-0.060 (-0.103, -0.018)		-0.052 (-0.092, -0.012)		-0.049 (-0.093, -0.004)		-0.079 (-0.123, -0.035)		-0.096 (-0.143, -0.050)	
Other	-0.122 (-0.503, 0.258)		-0.125 (-0.489, 0.239)		0.015 (-0.323, 0.353)		-0.179 (-0.567, 0.208)		-0.107 (-0.382, 0.167)	

The models were adjusted for BMI, age, sex, race, poverty income ratio, physical activity, total calcium, phosphorus, total protein, systolic blood pressure, diastolic blood pressure, and glycohemoglobin, except for the corresponding stratification variable. CI, confidence interval; BMD, bone mineral density.

Furthermore, sensitivity analyses demonstrated that these negative correlations remained robust even after adjusting for variables such as alanine aminotransferase, aspartate aminotransferase, alkaline phosphatase, blood urea nitrogen, and uric acid (**Supplementary Table S3**) or when the data were re-evaluated using unweighted methods (**Supplementary Table S4**).

## 4 Discussion

This study represents the investigation into the relationship between CMI and BMD. Utilizing data from the NHANES spanning 2005 to 2010, the research examined this association among the 12-19-year adolescent population. The findings indicated a correlation between CMI and reduced BMD. Furthermore, the analyses demonstrated that this relationship was consistent across various subgroups and remained unaffected by other confounding factors.

The relationship between obesity and bone health has garnered considerable attention and debate among scholars, yet controversy remains (14, 15). BMI is often used to evaluate obesity’s influence on BMD. Some studies show an inverted U-shaped association between BMI and BMD (16, 17). While other evidence indicates a negative correlation between BMI and BMD, suggesting that obesity may adversely affect bone health (18). Obesity is not just about weight gain; it includes fat accumulation and distribution. Individuals who are obese but have a normal BMI yet a high body fat percentage face a higher risk of lower BMD (19). We hypothesize that adipose tissue significantly influences the relationship between obesity and BMD.

Researchers have developed new indexes to examine the relationship between obesity and BMD by considering the impact of adipose tissue. The weight-adjusted waist index standardizes waist circumference and body weight, reflecting the level of central obesity and abdominal fat accumulation in obese individuals (20, 21). Similarly, the body roundness index and abdominal obesity index were used to assess the effects of central obesity and visceral fat on BMD (22, 23). The results of these studies align with the present findings, suggesting a link between obesity and fat accumulation and decreased BMD. However, additional research is needed to clarify the potential relationship between quantitative adiposity, dyslipidemia, and BMD. In this paper, we introduce the CMI index to more comprehensively evaluate the effects of obesity and lipids on BMD. The CMI, which reflects both fat distribution and lipid levels, presents a novel lens through which to examine these relationships. CMI can serve as a potential indicator for evaluating the impact of metabolic disorders on BMD, as it reflects cardiac metabolic burden.

The comparison with traditional lipid indicators suggests that CMI may be a more robust indicator of BMD, particularly at the femoral neck and lumbar spine (L1-L4), where it exhibited a stronger negative association than traditional variables. This aligns with previous findings that link metabolic dysregulation to bone health. However, in some skeletal sites (e.g., total femur and intertrochanter), certain traditional lipid markers showed significant associations, while CMI did not reach statistical significance. This may be attributed to differences in bone composition, metabolic activity, and potential confounding effects. Future studies should explore its potential utility alongside traditional lipid markers to better understand metabolic influences on bone health.

Multiple mechanisms may elucidate the impact of CMI on BMD. Obesity is frequently associated with systemic inflammation, triggering the release of pro-inflammatory factors and initiating inflammatory responses that can significantly contribute to decreased BMD (24, 25). Chronic low-grade inflammation associated with obesity may serve as a key mediator in the relationship between cardiometabolic health and bone metabolism. Adipose tissue secretes pro-inflammatory cytokines, such as tumor necrosis factor-alpha and interleukin-6, which can stimulate osteoclast activity and bone resorption while inhibiting osteoblast function. Furthermore, adipokines such as leptin and adiponectin have been implicated in bone remodeling and may partly explain the inverse association between CMI and BMD observed in this study (5). Gut dysbiosis, common in obese individuals, can further influence BMD (26). Moreover, vitamin D, crucial for maintaining BMD, is often found at lower levels in obese individuals due to sequestration in adipose tissue, potentially diminishing bone strength. Reduced growth hormone production in obese individuals may also contribute to lower BMD (27). Furthermore, obese individuals typically produce less growth hormone, which may contribute to lower BMD (28). Obesity-related oxidative stress can further reduce BMD by disrupting bone homeostasis and enhancing bone resorption (29–31). Additionally, dysregulated lipid metabolism also significantly impacts bone homeostasis, contributing to low BMD. Dyslipidemia-induced oxidative stress and inflammation promote increased osteoclast activity and bone resorption (32). Specifically, elevated cholesterol levels modulate the function of bone-resident osteoblasts and osteoclasts, accelerating bone deterioration (33). Furthermore, reduced HDL levels are linked to the development of an inflammatory microenvironment, impairing osteoblast differentiation and function. Perturbations in HDL metabolic pathways also favor adipoblastic differentiation while inhibiting osteoblastic differentiation, potentially through the modification of specific bone-related chemokines and signaling cascades (34). Collectively, these mechanisms may contribute to the observed negative association between CMI and BMD.

This is despite recent articles exploring the association between CMI and BMD, which have reached conflicting conclusions about CMI’s effect on femoral versus vertebral BMD (35, 36). We are still dedicated to utilizing CMI to investigate how childhood and adolescent obesity affects BMD. The two existing studies on CMI and BMI involved adult subjects, but there is a significant age difference among the participants, which may explain the contrasting findings. BMD and bone health are vital for the growth and development of young people, yet research on the effects of obesity during these stages is limited and inconsistent. Our study indicates that elevated lipid levels and obesity correlate with lower BMD and poorer bone health, highlighting the need to address metabolic disorders in children and adolescents. Notably, the seemingly paradoxical finding that BMD in the Q4 group was higher than in the Q1 group despite an overall negative correlation between CMI and BMD may be attributed to BMI confounding. Higher BMI is associated with both increased CMI and greater mechanical loading on bones, which may partially mask the negative metabolic effects of CMI on bone density.

Of course, there is no denying that this article has some shortcomings. As a cross-sectional investigation, it does not provide conclusive evidence for the causal association of CMI and BMD, thereby necessitating further studies to establish such links. Additionally, the geographic, demographic, and ethnographic constraints of this study may impact the generalizability of its findings. Another limitation of this study is the potential for measurement error in key variables (such as BMD and biochemical markers) due to variations in calibration or operator technique, despite the implementation of standardized protocols, and future studies should aim to improve the accuracy of these measurements. While the results offer valuable insights, these limitations must be considered when deciphering the significance of the research.

## 5 Conclusion

Utilizing data from the NHANES, we identified a robust negative association between CMI and BMD. Notably, this relationship remained unaffected by other confounding factors. These findings suggest that CMI may be a relevant factor associated with bone health in adolescents. Further research, particularly longitudinal studies, is needed to clarify potential underlying mechanisms.

## Data Availability

The raw data supporting the conclusions of this article will be made available by the authors, without undue reservation.
