# Supplementary material for: Association between cardiometabolic index and bone mineral density among adolescents in the United States
Source: Front Endocrinol (Lausanne). 2025 May 8;16:1535509. doi: 10.3389/fendo.2025.1535509 (PMC12095025; doi:10.3389/fendo.2025.1535509)
Supplement: Supplementary file 1 [file DataSheet1.pdf]

Supplementary material

Table S1 Category and definition of covariates.

Variable	Category and definition
Race	Non-Hispanic White, Non-Hispanic Black, Hispanic, and Other
Poverty income ratio	<1.3, 1.3-3.5, and >3.5
Physical activity	Physical activity is evaluated in terms of metabolic equivalent of task (MET), taking into account the duration, frequency, and intensity of vigorous and moderate exercise during leisure time each week, and then divided into low (<600 MET-min/week), medium (600-<1200 MET-min/week), and high (≥1200 MET-min/week)
Body mass index	Body mass index is calculated by dividing weight (in kilograms) by the square of height (in meters)

Table S2. Association between cardiometabolic index and traditional lipid indicators and bone mineral density.

Variable	***β*** **(95% CI)**	***P*** **value**
Total femur BMD		
CMI	-0.035 (-0.076, 0.005)	0.086
HDL-C	-0.028 (-0.060, 0.004)	0.083
LDL-C	-0.024 (-0.034, -0.014)	<0.001
LDL-C/HDL-C	-0.015 (-0.028, -0.003)	0.017
TC	-0.024 (-0.033, -0.015)	<0.001
Femoral neck BMD		
CMI	-0.052 (-0.087, -0.018)	0.004
HDL-C	-0.001 (-0.032, 0.031)	0.963
LDL-C	-0.024 (-0.031, -0.016)	<0.001
LDL-C/HDL-C	-0.021 (-0.031, -0.010)	<0.001
TC	-0.021 (-0.029, -0.013)	<0.001
Trochanter BMD		
CMI	-0.033 (-0.070, 0.004)	0.082
HDL-C	-0.028 (-0.055, -0.001)	0.045
LDL-C	-0.022 (-0.031, -0.012)	<0.001
LDL-C/HDL-C	-0.014 (-0.025, -0.003)	0.014
TC	-0.022 (-0.031, -0.014)	<0.001
Intertrochanter BMD		
CMI	-0.031 (-0.078, 0.015)	0.183
HDL-C	-0.039 (-0.077, -0.002)	0.042
LDL-C	-0.025 (-0.037, -0.012)	<0.001
LDL-C/HDL-C	-0.014 (-0.029, 0.002)	0.078
TC	-0.026 (-0.036, -0.015)	<0.001
Lumbar spine (L1-L4) BMD		
CMI	-0.048 (-0.085, -0.011)	0.013
HDL-C	-0.014 (-0.042, 0.014)	0.314
LDL-C	-0.022 (-0.032, -0.011)	<0.001
LDL-C/HDL-C	-0.017 (-0.028, -0.006)	0.004
TC	-0.021 (-0.030, -0.012)	<0.001

The models were adjusted for BMI, age, sex, race, poverty income ratio, physical activity, total calcium, phosphorus, total protein, systolic blood pressure, diastolic blood pressure, and glycohemoglobin. CI, confidence interval; BMD, bone mineral density.

Table S3. Association between cardiometabolic index and bone mineral density after additional adjustments for alanine aminotransferase, aspartate aminotransferase, alkaline phosphotase, blood urea nitrogen, and uric acid.

Variable	*β* (95% CI)	*P* value
Total femur BMD		
Continuous variable	-0.034 (-0.074, 0.007)	0.100
Categorical variable		
Q1	Reference	
Q2	-0.006 (-0.032, 0.019)	0.599
Q3	-0.012 (-0.036, 0.012)	0.311
Q4	-0.020 (-0.053, 0.013)	0.224
Femoral neck BMD		
Continuous variable	-0.051 (-0.086, -0.017)	0.005
Categorical variable		
Q1	Reference	
Q2	0.001 (-0.024, 0.027)	0.913
Q3	-0.012 (-0.037, 0.013)	0.336
Q4	-0.030 (-0.058, -0.002)	0.035
Trochanter BMD		
Continuous variable	-0.031 (-0.068, 0.005)	0.092
Categorical variable		
Q1	Reference	
Q2	-0.003 (-0.028, 0.021)	0.774
Q3	-0.006 (-0.026, 0.014)	0.551
Q4	-0.017 (-0.045, 0.010)	0.207
Intertrochanter BMD		
Continuous variable	-0.029 (-0.076, 0.018)	0.215
Categorical variable		
Q1	Reference	
Q2	-0.008 (-0.036, 0.020)	0.560
Q3	-0.013 (-0.041, 0.014)	0.332
Q4	-0.017 (-0.056, 0.022)	0.372
Lumbar spine (L1-L4) BMD		
Continuous variable	-0.045 (-0.081, -0.008)	0.018
Categorical variable		
Q1	Reference	
Q2	-0.012 (-0.036, 0.011)	0.293
Q3	-0.024 (-0.044, -0.004)	0.020
Q4	-0.033 (-0.062, -0.004)	0.027

The models were adjusted for BMI, age, sex, race, poverty income ratio, physical activity, total calcium, phosphorus, total protein, systolic blood pressure, diastolic blood pressure, glycohemoglobin, alanine aminotransferase, aspartate aminotransferase, alkaline phosphotase, blood urea nitrogen, and uric acid. CI, confidence interval; BMD, bone mineral density.

Table S4. Association between cardiometabolic index and bone mineral density using unweighted data.

Variable	*β* (95% CI)	*P* value
Total femur BMD		
Continuous variable	-0.050 (-0.080, -0.021)	<0.001
Categorical variable		
Q1	Reference	
Q2	-0.002 (-0.020, 0.016)	0.817
Q3	-0.009 (-0.027, 0.010)	0.366
Q4	-0.026 (-0.047, -0.005)	0.015
Femoral neck BMD		
Continuous variable	-0.054 (-0.082, -0.025)	<0.001
Categorical variable		
Q1	Reference	
Q2	0.005 (-0.012, 0.023)	0.549
Q3	-0.005 (-0.023, 0.014)	0.611
Q4	-0.025 (-0.045, -0.004)	0.018
Trochanter BMD		
Continuous variable	-0.049 (-0.076, -0.021)	<0.001
Categorical variable		
Q1	Reference	
Q2	-0.006 (-0.023, 0.011)	0.475
Q3	-0.008 (-0.026, 0.009)	0.342
Q4	-0.029 (-0.048, -0.009)	0.004
Intertrochanter BMD		
Continuous variable	-0.050 (-0.083, -0.016)	0.004
Categorical variable		
Q1	Reference	
Q2	-0.001 (-0.021, 0.020)	0.936
Q3	-0.007 (-0.029, 0.014)	0.496
Q4	-0.025 (-0.048, -0.001)	0.045
Lumbar spine (L1-L4) BMD		
Continuous variable	-0.062 (-0.089, -0.036)	<0.001
Categorical variable		
Q1	Reference	
Q2	-0.003 (-0.019, 0.014)	0.745
Q3	-0.017 (-0.034, 0.000)	0.046
Q4	-0.033 (-0.052, -0.014)	<0.001

The models were adjusted for BMI, age, sex, race, poverty income ratio, physical activity, total calcium, phosphorus, total protein, systolic blood pressure, diastolic blood pressure, and glycohemoglobin. CI, confidence interval; BMD, bone mineral density.

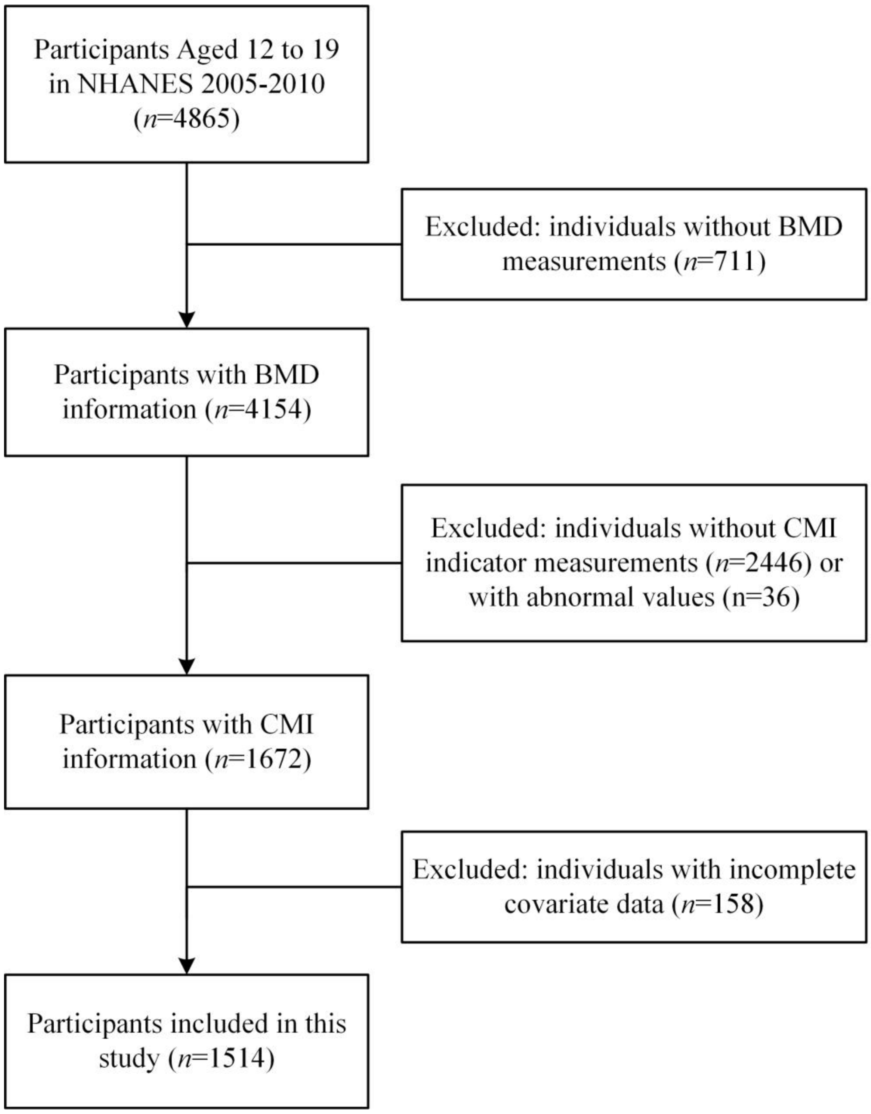

Figure S1. Flow diagram of the study participants included in NHANES 2005-2010.
